# Genetic and clinical peculiarities in a new family with hereditary hypophosphatemic rickets with hypercalciuria: a case report

**DOI:** 10.1186/1750-1172-5-1

**Published:** 2010-01-14

**Authors:** Natalia Mejia-Gaviria, Helena Gil-Peña, Eliecer Coto, Teresa M Pérez-Menéndez, Fernando Santos

**Affiliations:** 1 Hospital Universitario Fundación Santafé de Bogotá. Bogotá, Colombia; 2 Hospital Universitario Central de Asturias, Oviedo, Asturias, Spain; 3 Hospital Valle del Nalón. Langreo, Asturias, Spain; 4 Universidad de Oviedo. Oviedo, Asturias, Spain

## Abstract

Hereditary hypophosphatemic rickets with hypercalciuria is a rare autosomal recessive disorder (OMIM #241530), characterized by decreased renal phosphate reabsorption that leads to hypophosphatemia, rickets, and bone pain; hypophosphatemia is believed to stimulate 1,25 dihydroxyvitamin D synthesis which, in turn, results in hypercalciuria. Hereditary hypophosphatemic rickets with hypercalciuria is caused by loss-of-function in the type 2c sodium phosphate cotransporter encoded by the SLC34A3 gene. This report shows a family with a non-previously identified mutation in the SLC34A3 gene and exhibiting mild and different manifestations of HHRH. The probandus had hypophosphatemia, elevated serum 1,25 dihydroxyvitamin D concentrations, high serum alkaline phosphatase levels, hypercalciuria and nephrocalcinosis. The other members of the family presented some of these alterations: the mother, hypercalciuria and high 1,25 dihydroxyvitamin D concentrations; the son, hypercalciuria, high 1,25 dihydroxyvitamin D values and elevated alkaline phosphatases; the father, high alkaline phosphatases. The genetic analysis revealed the existence of a single mutation (G78R) in heterozygosis in the SLC34A3 gene in the probandus, her mother and her brother, but not in the father. These findings suggest that he mutation in heterozygosis likely gave rise to a mild phenotype with different penetrance in the three relatives and also indicates that the elevation of 1,25 dihydroxyvitamin D does not result from hypophosphatemia. Thus, this family raises some issues on the transmission and pathophysiology of hereditary hypophosphatemic rickets with hypercalciuria.

## Background

Hereditary hypophosphatemic rickets with hypercalciuria (HHRH) is a rare autosomal recessive disorder (OMIM #241530), characterized by decreased renal phosphate reabsorption that causes hypophosphatemia, rickets, and bone pain. Hypophosphatemia stimulates the renal 1a-hydrolase, increasing in turn the synthesis and serum levels of 1,25 dihydroxyvitamin D, with the resultant suppression of parathyroid hormone (PTH) levels, increased intestinal reabsorption of calcium and hypercalciuria, which differentiates HHRH from autosomal dominant hypophosphatemic rickets (ADHR) and X-linked hypophosphatemia (XLH).

Tieder et al. [1] described in 1985 a consanguineous Bedouin kindred in which 6 members had hypophosphatemic rickets and hypercalciuria, probably the same disorder reported by Royer [2] in 1962 as hypercalciuric rickets. Bergwitz et al. [3] performed a genomewide linkage scan combined with homozygosity mapping in the large consanguineous Bedouin kindred reported by Tieder et al [1]. The candidate gene region was narrowed to a 1.6 Mb region on chromosome 9q34, and the mutated gene was identified as the SLC34A3, which encodes the type 2c sodium phosphate cotransporter (NPT2c), that, with the NPT2a, is expressed at the apical domain of renal proximal tubular cells and reabsorb phosphate from the glomerular filtrate under the hormonal control of PTH and the fibroblast growth factor 23 (FGF23) [4-7].

HHRH has been defined as an autosomal recessive disorder because obligate carriers of the mutation, such as the parents of affected children, have borderline hypercalciuria but not hypophosphatemia or rickets [8]. However, because of the low prevalence of the disease, the mode of inheritance has been inferred from a limited number of families worldwide. From its initial description in 1985, few families [1,8-10] and sporadic cases [11-14] have been reported in Turkey, Holland, Morocco, North America, Japan and Germany.

## Case report

**Figure 1:**
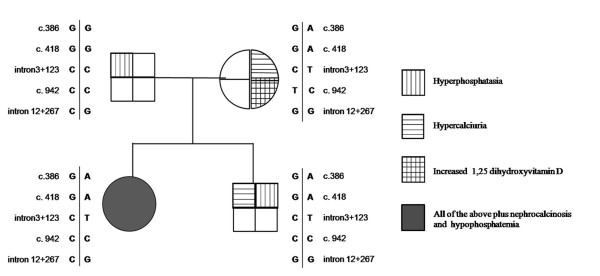
**Family pedigree showing the haplotypes and analytical findings**. In addition to the c.418 G>A mutation, the genotypes for the intron 3+c.123 C>T, exon 8 c.942 T>C, and intron 12+c.267 G>C are also indicated.

A four member Spanish family is described in this report (Figure 1). Parents were not consanguineous and all members were asymptomatic. The daughter was referred to our outpatient clinic at the age of 11 years for evaluation of hyperphosphatasia and hypercalciuria. She had been suffering of intermittent abdominal pain for 3 years but denied bone pain or renal colic. At the first evaluation her height was 153.3 cm (50^th ^percentile) and her weight 44.5 kg (50^th ^percentile), normal blood pressure (116/60 mm Hg) and the only abnormal finding in the physical examination was a mild dorsal scoliosis. The initial serum biochemistry in our clinic confirmed the previous biochemical alterations and showed decreased tubular reabsorption of phosphate (TRP) and high circulating levels of 1,25 dihydroxyvitamin D. Acid-base status and serum urea, creatinine, albumin, lipid profile and liver-function tests were normal. Bilateral medullar nephrocalcinosis was found by renal ultrasonography. No radiological signs of rickets were present, bone mineral density was within normal reference values and bone age was according to chronological age. Oral phosphate supplementation was prescribed but only for a short period because of patient's bad compliance and lack of major disease-related manifestations. Significant biochemical findings of the patient and her family are shown in Table 1. Renal ultrasound of the parents and brother were normal. After a 7.5 year follow up, the four family members continue asymptomatic and their initial laboratory alterations remain essentially unchanged.

**Table 1:** Initial/final significant findings in the family

	Patient	Mother	Father	Brother
**Age at diagnosis **(years)	11	33	41	1
**Serum alkaline phosphatase **(Times above the upper limit of normal reference values)	4.0/2.0	0.6/1.1	2.5/1.5	1.5/3.5
**Serum calcium **(mg/dl) (Normal reference values: 8.5-10.5)	10.5/9.8	9.46/9.84	10.0/8.8	10.6/10.3
**Serum phosphate **(mg/dl)	3.2/2.3	2.6/4.3	4.2/2.8	5.3/5.0
**Urine calcium/creatinine **(mg/mg) (Normal reference values: <0.20)	0.45/0.21	0.33/0.27	0.17/0.04	0.52/0.34
**Urine calcium **(mg/kg/day) (Normal reference values: < 4.0)	9/5	6.4/NA	4.5/NA	5.5-6.2
**Urine phosphate **(mg/kg/day) (Normal reference values: 12.4 ± 4.6)	28.0/16.9	12.5/NA	16.1/NA	28.0/NA
**Tubular reabsorption of phosphate (%) **(Normal reference values: 75-85)	63.8/74.4	79.0/90.5	77.8/NA	78.1/89.9
**TmP/GFR **(mg/dl) (Normal reference values: 2.5-4.2)	2.1/1.8	2.1/4.2	3.3/NA	4.1/4.9
**Serum intact PTH **(pg/ml) (Normal reference values:10-65)	9/22	62/27	38/41	13/13
**Serum 25 hydroxivitamin D **(ng/ml) (Normal reference values: 10-68)	16/18	18/20.4	41/24.2	29/29
**1,25 dihydroxyvitamin D **(pg/ml) (Normal reference values: 20-63)	109/96	83/80	37/38	71/64
**eGFR **(ml/min/1.73 m^2^)	104/109	83/107	84/101	144/159

Normal values for serum phosphorus: < 14 years: 3.5-6.5 mg/dl; Adults: 2.7-4.5 mg/dl. TmP/GFR values were calculated by the Walton Bijvoen nomogram. [16]. eGFR: Estimated glomerular flow rate based on Cockroft-Gault formula for adults and Schwartz formula for children. NA: not available.

The study was approved by the Ethical Committee of our institution. The two parents gave their informed consent to participate in the study, including the genetic study of their children. Genomic DNA was obtained from leukocytes in 10 ml of blood, and the 13 coding exons and intronic flanking nucleotides of the *SLC34A3 *gene were amplified by polymerase chain reaction (PCR). These PCR fragments were purified and both DNA strands were sequenced on an automated ABI310 system (Applied Biosystems - Applera Corporation, Drive Foster City, CA, USA).

We found a nucleotide change in exon 4 (c.418 G>A) that results in a missense amino acid change, Glycine78Arginine (G78R). This has not been reported as a SLC34A3 polymorphism in the genome database http://www.ensembl.org, and the glycine 78 was conserved among species. The c.418 A created a site for the restriction enzyme *PstI *(CTGCA/G), giving two fragments of 117 + 354 bp whereas the wild type allele was seen as a single fragment of 471 bp. We confirmed the absence of this putative mutation in 200 healthy controls by digestion of PCR fragment with PstI, followed by electrophoresis on 3% agarose gels. The patient, her mother and her brother were heterozygous for this mutation and the father was wild-type homozygote.

In addition to G78R, the patient was heterozygous for several polymorphisms (Figure 1). The patient was homozygous for a polymorphism in the last nucleotide of exon 8 (c.942 T>C). The patient's mother was heterozygous carrier for this exon 8 variant, while the father and brother were also c.942 C homozygotes. Although this change does not change the amino acid (L253L), *in silico *analysis with the BDGP program http://www.fruitfly.org/seq_tools/splice.html predicts an effect on pre-mRNA splicing as a result of a reduction of the score for the splicing (0.64 vs. 0.54) at the exon 8-intron 8 boundary. Thus, the allele c.942 C might affect splicing, skip exon 8 and produce an aberrant mRNA. To assess the potential effect on splicing, mRNA of the proband, their parents and brother and two healthy donors, was obtained from peripheral leukocytes. The cDNA was synthesized and PCR amplified with primers that matched exons 3 and 9. We were unable to obtain amplified product from any of the cDNAs, including the two controls, and could not determine the putative effect of this variant on pre-mRNA splicing.

## Conclusions

This family presents phenotypic and genetic peculiarities that deserve some comments. The mother and her two siblings were heterozygous for a non-previously identified missense mutation in the *SLC34A3 *gene (c.418 G>A; G78R). The amino acid 78 of NPT2c is conserved among species (glycine) and is located in the first membrane-spanning domain of the cotransporter. The glycine to arginine change likely disrupts the transmembrane domain, resulting in an abnormal transporter channel function responsible for the clinical manifestations. The other nucleotide changes found in these subjects correspond to polymorphisms. Unfortunately, and likely as a result of the low expression of NPT2c in leukocytes, we did not succeed in studying the potential impact of these polymorphisms on RNA splicing. The daughter had nephrocalcinosis, hypophosphatemia, hypercalciuria and elevated serum 1,25 dihydroxyvitamin D concentrations whereas her mother and brother, in spite of carrying the same G78R mutation, only had hypercalciuria and increased 1,25 dihydroxyvitamin D. This indicates either the girl, and likely the father, carried a second mutation out of the region analyzed, for instance, in the promoter or intronic nucleotides that were not sequenced or the mutation in heterozygosis gave rise to a mild phenotype with different penetrance in the three relatives. The last one is the most plausible hypothesis because heterozygous SLC34A3 mutations leading to a variety of biochemical abnormalities have already been reported in previous HHRH families [3]. The elevation of alkaline phosphatases in the father likely corresponds to benign hyperphosphatasemia [15], because of the absence of symptoms and the lack of associated biochemical alterations, although accounted for an additional source of confusion in the interpretation of the clinical picture of the family.

It is also of note that the mother and his son were hypercalciuric and had high levels of 1,25 dihydroxyvitamin D, particularly the mother, in the presence of normal serum concentrations of phosphate. This challenges the pathophysiological concept that in HHRH hypophosphatemia triggers the increased production of 1,25 dydroxyvitamin D which, in turn, causes hypercalciuria. Our findings support the relationship between elevations of 1,25 dihydroxyvitamin D and urinary calcium but not the hypophosphatemia as the primary event responsible for these alterations. It could be hypothesized that downregulation of FGF23 serum levels, due to decreased phosphate reabsorption, could explain, at least partly, the elevation of the 1-25 vitamin D. Unfortunately, FGF23 was not measured.

In summary, this report describes an additional family with HHRH, finds a new mutation in the SLC34A3 gene and, more interestingly, raises some issues on the genetic basis of this disease and its pathophysiological mechanism.

## Consent

Written informed consent was obtained from the patient for publication of this case report and any accompanying images. A copy of the written consent is available for review by the Editor-in-Chief of this journal.

## Competing interests

The authors declare that they have no competing interests.

## Authors' contributions

FS and MTPM were responsible for the diagnosis and clinical follow-up of this family. NMG and HGP wrote down the first draft of the manuscript. HGP and EC carried out the molecular genetic studies. All authors revised and approved the final version of the manuscript.

## References

[B1] Tieder M Modai D Samuel R Arie R Halabe A Bab I Gabizon D Liberman UA Hereditary hypophosphatemic rickets with hypercalciuria N Engl J Med 1985 312 611 617 2983203 10.1056/NEJM198503073121003

[B2] Royer P Mathieu H Gerbeaux S Frederich A Rodriguez-Soriano J Dartois AM Cuisinier P Idiopathic hypercalciuria with nanism and renal involvement in children Ann Pediatr (Paris) 1962 9 147 163 14494745

[B3] Bergwitz C Roslin NM Tieder M Loredo-Osti JC Bastepe M Abu-Zahra H Frappier D Burkett K Carpenter TO Anderson D Garabedian M Sermet I Fujiwara TM Morgan K Tenenhouse HS Juppner H SLC34A3 mutations in patients with hereditary hypophosphatemic rickets with hypercalciuria predict a key role for the sodium-phosphate cotransporter NaPi-IIc in maintaining phosphate homeostasis Am J Hum Genet 2006 78 179 192 10.1086/499409 16358214 PMC1380228

[B4] Segawa H Kaneko I Takahashi A Kuwahata M Ito M Ohkido I Tatsumi S Miyamoto K Growth-related renal type II Na/Pi cotransporter J Biol Chem 2002 277 19665 19672 10.1074/jbc.M200943200 11880379

[B5] Foster IC Hernando N Biber J Murer H Proximal tubular handling of phosphate: A molecular perspective Kidney Int 2006 70 1548 1559 10.1038/sj.ki.5001813 16955105

[B6] Tenenhouse HS Phosphate transport: Molecular basis, regulation and pathophysiology J Steroid Biochem Mol Biol 2007 103 572 577 10.1016/j.jsbmb.2006.12.090 17270430

[B7] Murer H Hernando N Forster I Biber J Proximal tubular phosphate reabsortion: Molecular Mechanisms Physiol Rev 2000 80 1373 1409 11015617 10.1152/physrev.2000.80.4.1373

[B8] Lorenz-Depiereux B Benet-Pages A Eckstein G Tenenbaum-Rakover Y Wagenstaller J Tiosano D Gershoni-Baruch R Albers N Lichtner P Schnabel D Hochberg Z Strom TM Hereditary hypophosphatemic rickets with hypercalciuria is caused by mutations in the sodium-phosphate cotransporter gene SLC34A3 Am J Hum Genet 2006 78 193 201 10.1086/499410 16358215 PMC1380229

[B9] Tieder M Modai D Shaked U Samuel R Arie R Halabe A Maor J Weissgarten J Averbukh Z Cohen N Edelstein S Liberman UA 'Idiopathic' hypercalciuria and hereditary hypophosphatemic rickets: two phenotypical expressions of a common genetic defect New Eng J Med 1987 316 125 129 3796683 10.1056/NEJM198701153160302

[B10] Heuvel L van de de Koul KO Knots E Knoers N Monnens L Autosomal recessive hypophosphataemic rickets with hypercalciuria is not caused by mutations in the type II renal sodium/phosphate cotransport gene Nephrol Dial Transplant 2001 16 48 51 10.1093/ndt/16.1.48 11208993

[B11] Kremke B Bergwitz C Ahrens W Schütt S Schumacher M Wagner V Holterhus PM Jüppner H Hiort O Hypophosphatemic rickets with hypercalciuria due to mutation in SLC34A3/NaPi-IIc can be masked by vitamin D deficiency and can be associated with renal calcifications Exp Clin Endocrinol Diabetes 2009 117 49 56 10.1055/s-2008-1076716 18523928 PMC4777409

[B12] El Aichaoui S Bahiri R Mahfoud S Benbouazza K Guédira N Hajjaj-Hassouni N Hereditary hypophosphatemic rickets with hypercalciuria Joint Bone Spine 2006 73 481 482 10.1016/j.jbspin.2005.11.019 16798045

[B13] Ichikawa S Sorenson AH Imel EA Friedman NE Gertner JM Econs MJ Intronic deletions in the SLC34A3 gene cause hereditary hypophosphatemic rickets with hypercalciuria J Clin Endocrinol Metab 2006 91 4022 4027 10.1210/jc.2005-2840 16849419

[B14] Jaureguiberry G Carpenter TO Forman S Jüppner H Bergwitz C A novel missense mutation in SLC34A3 that causes hereditary hypophosphatemic rickets with hypercalciuria in humans identifies threonine 137 as an important determinant of sodium-phosphate cotransport in NaPi-IIc Am J Physiol Renal Physiol 2008 295 369 370 10.1152/ajprenal.00090.2008 PMC2519180 18480181

[B15] Wilson JW Inherited elevation of alkaline phosphatase activity in the absence of disease N Engl J Med 1979 301 983 4 492229 10.1056/NEJM197911013011806

[B16] Walton RJ Bijvoet OL Nomogram for derivation of renal threshold phosphate concentration Lancet 1975 16 2 309 10 10.1016/S0140-6736(75)92736-1 50513

